# A Retrospective Study of Postmastectomy Pain Syndrome: Incidence, Characteristics, Risk Factors, and Influence on Quality of Life

**DOI:** 10.1155/2013/159732

**Published:** 2013-11-27

**Authors:** Yang Meijuan, Peng Zhiyou, Tang Yuwen, Feng Ying, Chen Xinzhong

**Affiliations:** ^1^Department of Anesthesiology, Women's Hospital, School of Medicine, Zhejiang University, No. 2 Xueshi Road, Hangzhou, Zhejiang 310006, China; ^2^Department of Anesthesiology, First Affiliated Hospital, School of Medicine, Zhejiang University, Hangzhou, Zhejiang 310006, China

## Abstract

*Objective*. The underlying cause for postmastectomy pain syndrome (PMPS) and its impact on quality of life remain unclear. The objective of this study aims to determine retrospectively the prevalence of PMPS, its predicting risk factors, and its impact on quality of life. *Method*. In this survey, 225 women completed a battery of questionnaires. The questionnaires comprised the short form of the McGill Pain Questionnaire (SF-MPQ) exploring the characteristics and the description of the pain, and a Short Form-36 (SF-36) Health Survey evaluating quality of life. Logistic regression analyses were subsequently performed to identify risk factors for PMPS. *Results*. 62 women (27.6%) reported PMPS as a consequence of surgery, and the pain was generally mild, mostly localized in breast area and intermittent. The pain was mainly described as aching (62.9%). 144 women reported sensory disturbance. We found that only the younger age is the predictive factor for PMPS (*P* < 0.05). Compared to the patients who did not experience PMPS, those who suffered from PMPS had significantly worse scores in role limitations due to physical problems (role physical, RP), body pain (BP), general health (GH), vitality (VT), role limitations due to emotional problems (role emotional, RE), and mental health (MH) (*P* < 0.05). *Conclusion*. PMPS is a significant problem, and the possible risk factors should be further explored. Patients with PMPS have significant worse quality of life, suggesting that patients should be well informed about the likelihood of experiencing the pain, and they may be afforded greater predictability and higher perceived control to enhance their quality of life.

## 1. Introduction

It is reported that postmastectomy pain syndrome (PMPS) is a common problem, ranging from 25% to 60% [1, 2]. The pain is localized in the axilla, medial upper arm, breast, and/or chest wall and lasting beyond three months after surgery when all other causes of pain such as infection have been eliminated [3]. The pain seriously affects the patient's mood, everyday activities, and social function and causes heavy economic burden for the healthcare system [4, 5]. Several risk factors for the development of persistent postsurgical pain have been proposed, including adjuvant therapy, age, psychosocial status, preoperative breast pain, type of surgery, type of analgesia, and genetics [6, 7]. Being a problem of such magnitude, increasing attention should be paid to PMPS. The purpose of this retrospective study was to examine the prevalence, treatment-related and physiological factors associated PMPS in China. The secondary goal was to clarify severity of the pain and the impact of chronic pain on daily life.

## 2. Method

### 2.1. Subjects

A retrospective questionnaire survey was undertaken on patients who had undergone surgery for breast cancer from Jan 2010 to Dec 2011 in our hospital. Exclusion criteria were non-Chinese patients, benign breast pathology, surgical site infection, or death. The investigation was conducted between Jan 2013 and May 2013. The study was approved by ethics committees of Women's Hospital, School of Medicine, Zhejiang University, before initiating, and all subjects provided verbal informed consent.

### 2.2. Definitions and Questionnaire Design

According to the International Association for the Study of Pain (IASP) [8] and recent studies [3, 7], we defined PMPS as chronic pain in the anterior aspect of the thorax, axilla, and/or upper half of the arm beginning after mastectomy or quadrantectomy and persisting either continuously or intermittently for more than three months after the surgery, and the pain nature included numbness, pins and needles, burning, or stabbing. The pain caused by other reasons such as chronic infection should be excluded.

The questionnaires used consisted of two parts. The first part concerning pain characteristics assessment was modulated through the short form of the McGill Pain Questionnaire (SF-MPQ) [9]. The SF-MPQ describes the temporal characteristics of such pain, recognizing the time of appearance after surgery, the timing of occurrence. It includes body charts where participants drawed the location of the pain and described its characteristics dull, pins and needles, burning, stabbing, and ache. The second part covered quality of life and was assessed using the Short Form-36 (SF-36) [10]. The SF-36 assesses eight scales: physical function (PF), role limitations due to physical problems (role physical, RP), body pain (BP), general health (GH), vitality (VT), social function (SF), role limitations due to emotional problems (role emotional, RE), and mental health (MH). The raw scores of each subscale were converted to a range from 0 to 100: higher scores indicated better level of functioning or well-being. Both questionnaires had been previously used in breast cancer surgery research [4].

### 2.3. Study Procedures

Patients included in this study received a telephone interview to make the oral consent to participate in our questionnaires research. Informed consent was also requested for viewing the patients' medical records on the phone. Then, SF-MPQ with minor modifications and SF-36 (both in Chinese version) were mailed to patients who agreed to take part in the survey, and they were asked to mail back the questionnaires after questions were answered carefully. Those patients who failed to respond within four weeks were contacted by telephone to find out why and, if necessary, mailed the questionnaire again. Eight weeks after sending the first questionnaires, the investigation was closed.

### 2.4. Patient's Medical Record Collection

Patients' demographic and disease-related data were retrieved from the hospital and department database to identify possible risk factors contributing to chronic pain development. The data included age, BMI, presence of diabetes mellitus and hypertension, type of surgery, surgical complication (e.g., wound infections, secondary hemorrhage, and hematomas), preoperative and postoperative adjuvant therapies that included radiation therapy, chemotherapy, and hormonal therapy.

### 2.5. Statistical Analysis

Data was analyzed using SPSS 16.0 software (SPSS, Inc., Chicago, IL, USA). Continuous variables were presented as mean ± standard deviations, and categorical data were shown as numbers and percentages. Comparison of continuous data was performed by Student's *t*-test with normal distribution and by the Mann-Whitney *U* test for variables with nonnormal distribution. The Chi-square test was used to compare groups with categorical variables. *P* value less than 0.05 was considered significant. Univariate analyses and multiple forward stepwise logistic regression analyses were performed to identify risk factors predicting PMPS. After univariate analyses, variables with *P* value less than 0.05 were included in a multivariate logistic regression analysis to identify independent factors of PMPS.

## 3. Results

### 3.1. Sample Tracing Response Rate

349 patients were contacted through the phone, and 97 patients refused to participate. Among all these 252 returned mails, 27 patients returned the questionnaire but did not fill in the part of the questionnaire concerning pain or quality of life and were excluded from the analysis. Ultimately, we investigated and analyzed data from 225 patients.

### 3.2. Characteristics of Responders

225 questionnaires and their medical record were evaluable. Results showed that the average age was 53 years (range, 29 to 74 years) and average BMI of 23 kg/m^2^ (range, 16 to 31 kg/m^2^). Some of the patients were suffering from concomitant diseases: 49 (21.8%) were affected by hypertension, and 12 (5.3%) used oral antidiabetic drugs.

### 3.3. Pain

62 patients (27.6% of 225 patients) reported pain as a consequence of treatment. 50 patients (80.6% of 62 patients) reported mild pain, 10 patients (16.1%) developed moderate pain, and 2 patients (3.2%) developed severe pain. Of all the patients who developed pain, only 3 patients (4.8%) had taken oral analgesics. 35.5% experienced pain a few days after surgery, 25.8% patients developed pain a few weeks later, and 38.7% reported that pain started a few months later. Patients described frequency of pain following surgery: transient pain (*n* = 14, 22.6%), intermittent pain (*n* = 41, 66.1%), and continuous pain (*n* = 7, 11.3%). It was also shown that the specific location of pain could be chosen more than once, and the majority of patients chose the breast area and, secondly, the scar. A detailed description of the pain characteristics was shown in Table 1. In terms of the sensitive component of the SF-MPQ (Table 2), the most frequently selected terms were aching (62.9%), dull (48.4%), or pulling (27.4%). In the affective, components, the word “tiring” was most frequently chosen. The mean SF-MPQ scores for the sensitive, affective, and total components were 3.45, 1.53, and 4.98, respectively, and the mean of words chosen was 3.22.

### 3.4. Sensory Disturbance

**Figure 1:**
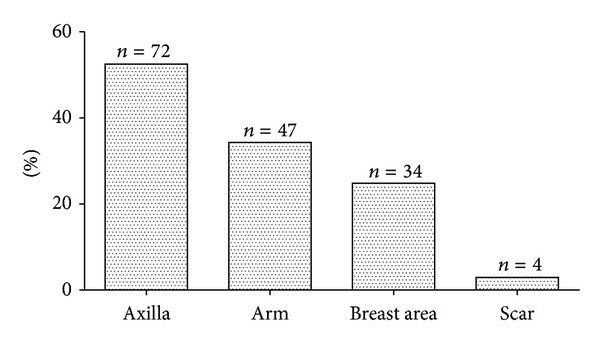
Location of sensory disturbance after breast cancer surgery. *n* = number of patients.

A total of 144 women (64%) reported sensory disturbances or discomfort after surgery. As shown in Figure 1, the most frequently involved areas were the axilla (*n* = 72, 52.5%), followed by arm (*n* = 47, 34.3%), breast area (*n* = 34, 24.8%), and the scar (*n* = 4, 2.9%). The most frequently described terms over the affected area were numbness (*n* = 98, 71.5%), pins-and-needles (*n* = 24, 17.5%), and loss of sensation (*n* = 17, 12.4%). A total of 47 women (32.6%) reporting sensory disturbances suffered pain as well compared with 97 (67.4%) reporting no pain, indicating that sensory disturbances may be an increased risk of chronic pain (*P* = 0.023). This strong association was not attributed to other variables on multivariate analysis.

### 3.5. Quality of Life

**Figure 2:**
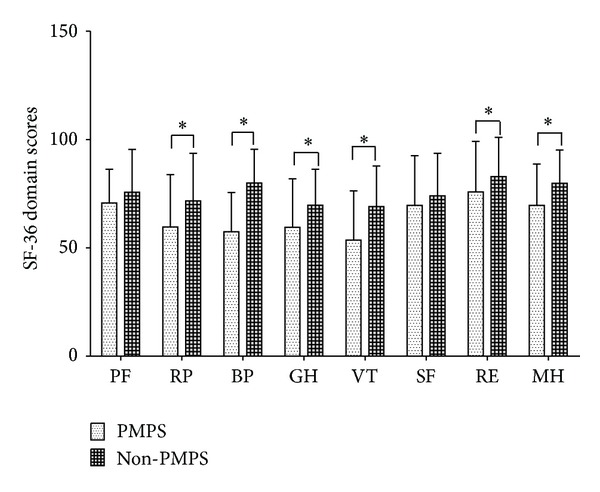
SF-36 domain scores of patients after breast surgery. Values of SF-36 domain scores represent mean ± SD in the respective group. PMPS, postmastectomy pain syndrome; Non-PMPS, no postmastectomy pain; PF, physical function; RP, role limitations due to physical problems; BP, body pain; GH, general health; VT, vitality; SF, social function; RE, role limitations due to emotional problems; MH, mental health. *Significant result, *P* < 0.05.

In order to assess the impacts of PMPS on quality of life in detail, SF-36 Health Survey was used. As shown in Figure 2, compared to patients who did not experience PMPS, patients with PMPS had significantly lower SF-36 scores across all health domains, except for physical function (PF) and social function (SF) (*P* < 0.05).

### 3.6. Risk Factors for PMPS

No significant association with the report of persistent pain was found in BMI (data not shown), type of surgery, and perioperative adjuvant therapy (Table 3) between patients with pain or without pain. Women with PMPS were younger than those without pain (50.5 ± 8.0 y versus 54.6 ± 9.9 y, *P* < 0.05), which implied that younger women tended to develop more pain after surgery.

## 4. Discussion

PMPS attracted considerable attention recently, but there is no agreement regarding the prevalence and risk factors. The aim of this retrospective study was to show the prevalence of PMPS in Zhejiang province of China. The result revealed that the incidence of PMPS was 27.6%, which was similar to recent studies [7, 11]. Although the chronic pain was generally mild, patients who developed PMPS were at risk for significantly decreased role limitations due to physical problems, body pain, general health, vitality, role limitations due to emotional problems, and mental health. Our results also suggested that women in younger age tended to develop more pain than women in older age.

The reported prevalence of PTPS varied from 25% to 60% [1, 2]. This wide interval may be explained by discrepancies in terms of definitions used to ascertain PMPS, timing of assessment, or the age group of the population studied. In this study, we used strict defining criteria to select eligible patients. The definition of PMPS we applied was the pain lasted for at least three months duration according to the IASP. We excluded nonneuropathic pain and pain outside the distribution of the nerves affected [3, 7].

Although prevalence of PMPS is high, this situation is rarely reported in our country, and it is still considered to be relatively uncommon. One explanation is that breast cancer is a potentially life threatening disease, then clinicians and patients mainly focus on the treatments while disregarding the PMPS. Furthermore, very few patients take the oral analgesia, although an increasing number of breast cancer survivors experience pain. This implied that doctors should follow up patients with emphasis not only on long and effective observation of the treatment, but also on noting any problems advent such as the pain during treatment to enhance the patients' quality of life. The pathogenic mechanisms of PMPS are multifactorial including tissue injury, nerve damage related to surgical procedures, and neuroma pain. Different types of sensory disturbances (e.g., numb, electric shock, distending, itchy, burning, or loss of sensation) are sequelae to surgery and may be an important part of the pain characteristics [12, 13].

Risk factors for the development of PMPS can be related to the patient or the surgery itself. Among patient-related factors, we find that the younger age was shown to be a predictive factor for the development of PMPS, and our results are consistent with previous literature [1, 14, 15]. Many explanations have been proposed, including (1) younger patients were subjected to more aggressive use of adjuvant therapies; (2) age related decrease in pain perception; (3) negative oestrogen receptor status; (4) increased nerve sensitivity; (5) greater surgical invasion in axillary dissection. For axillary clearance is more challenging because of more fatty tissue [16, 17], some researchers consider an elevated BMI as a risk factor for this syndrome, but our results showed no correlation.

In recent studies, axillary dissection (not the mastectomy or quadrantectomy) has been demonstrated to be a risk factor for persistent pain after treatment and sensory disturbances compared with sentinel lymph node biopsy [6, 7, 17]. Intercostobrachial nerve (ICBN) enters the axilla and passes through with considerable variability to the posteromedial border of the upper arm [18]. Thus, the ICBN is vulnerable during axillary surgery. When axillary is dissecting, ICBN which supplies sensation to the upper arm and shoulder is commonly injured. However, our study failed to prove this phenomenon.

Adjuvant radiotherapy has also been identified as a risk factor for the pain [15, 19], but this was not confirmed in our study. One reason for the difference may be our small sample size of patient selecting the radiotherapy. There was no previous study that indicated exact information about the dose and location of radiotherapy. Chemotherapy has not been shown to be a risk factor for PMPS in previous study [1, 7], and our result was consistent with this phenomenon. Neurotoxicty is a well-known attribute of many chemotherapeutic agents used in breast cancer treatment [19, 20]. In a recent study, researchers found that the neurotoxic chemotherapeutic agent docetaxel showed no influence on persistent pain in the area of surgery but a significant association with peripheral neuropathy [21]. Yet, more studies should be done to provide detail information regarding chemotherapeutic agent used, the cumulative dose, side effects, and the timing of the administration, to elucidate whether or not chemotherapy is a predictive factor for PMPS. In this study, no relation between endocrine therapy and PMPS was found. There are two parts in the endocrine therapy consisting of selective estrogen receptor modulators and aromatase inhibitors. Both groups are known to induce musculosueletal pain, so they can confuse our understanding on pathophysiological mechanisms of PMPS [22]. Therefore, more research should be carried out to find out what role they play in the treatment.

Based on SF-36 scores, our study has found that patients' subjective reports of pain were significantly related to quality of life. Women with PMPS had lower SF-36 scores across all health domains, except for physical function and social function, than women who did not develop pain. Our results suggested that in patients who suffer pain after surgery, psychophysical adaptation was decreased compared to patients without pain, which was consistent with previous findings [23, 24].

PMPS is a common complication following breast surgery, which could significantly impact patient's daily life in a negative way. Our study has demonstrated that younger age is an independent risk factor for PMPS. More detailed prospective studies are required to identify the risk factors of PMPS and to plan possible prevention. Meantime, surgeons should keep good communication with patients who underwent breast surgery on the possibility of developing chronic pain to help them alleviate the distress.

## Figures and Tables

**Table 1:** Pain characteristics.

	*n*	%
Pain occurrence		
Yes	62	27.6
No	163	80.6
Pain start		
A few days	22	35.5
A few weeks	16	25.8
A few months	24	38.7
Pain location		
Breast area	37	59.7
Scar	29	46.8
Arm	17	27.4
Axilla	5	8.1
Back	3	4.8
Pain timing		
Transient	14	22.6
Intermittent	41	66.1
Continuous	7	11.3
Pain severity		
Mild	50	80.6
Moderate	10	16.1
Severe	2	3.2

**Table 2:** Percentage of patients experiencing pain who selected a term to describe it.

Descriptive terms	*n*	%
Sensory		
Aching	39	62.9
Dull	30	48.4
Pulling	17	27.4
Stabbing	7	11.3
Splitting	5	8.1
Burning	11	17.1
Gnawing	16	25.8
Sharp	8	12.9
Shoot	5	8.1
Throbbing	3	4.8
Affective		
Tiring, exhausting	27	43.5
Fearful	21	33.9
Punishing, cruel	7	11.3
Sickening	4	6.5

**Table 3:** Risk factors for PMPS.

	PMPS (%)	No PMPS (%)	*P*
Overall (*n* = 225)			
Mastectomy (*n* = 220)	61 (27.1)	159 (70.7)	0.702
Breast conservative surgery (*n* = 5)	1 (0.4)	4 (1.8)	
Overall (*n* = 225)			
No lymph nodes dissection (*n* = 13)	4 (1.8)	9 (4)	0.960
Sentinel lymph nodes sampling (*n* = 14)	4 (1.8)	10 (4.4)	
Axillary dissection (*n* = 198)	54 (24)	144 (64)	
Preoperative radiotherapy (*n* = 1)	0 (0)	1 (100)	0.536
Postoperative radiotherapy (*n* = 6)	1 (16.7)	5 (83.3)	0.545
Preoperative chemotherapy (*n* = 23)	4 (17.4)	19 (82.6)	0.250
Postoperative chemotherapy (*n* = 184)	51 (27.7)	133 (72.3)	0.908
Sensory disturbance (*n* = 144)	47 (32.6)	97 (67.4)	0.023*

*Significant result, *P* < 0.05.
